# Characteristics and Phylogeny of *Shewanella haliotis* Isolated from Cultivated Shellfish in Taiwan

**DOI:** 10.1155/2018/9895148

**Published:** 2018-07-09

**Authors:** Zong-Yen Wu, Po-Yu Liu, Shu-Ying Tseng, Yi-Hsuan Lee, Shu-Peng Ho

**Affiliations:** ^1^Department of Veterinary Medicine, College of Veterinary Medicine, National Chung-Hsing University, Taichung, Taiwan; ^2^Department of Internal Medicine, Taichung Veterans General Hospital, Taichung, Taiwan

## Abstract

*Shewanella haliotis* is an emerging human pathogen. Many infectious cases were linked to shellfish ingestion or aquatic exposure. Therefore, it is important to study the phylogeny and distribution of *S. haliotis* in shellfish aquaculture. We investigated the distribution of *S. haliotis* in cultivated shellfish farming in Taiwan in which *S. haliotis* was found in the shellfish from all sampling sites. *S. haliotis* was identified in cultivated shellfish by 16S rRNA gene sequencing, such as abalone (*Haliotis diversicolor*), clam (*Meretrix lusoria*), and oyster (*Crassostrea gigas*). This study highlighted the contamination of *S. haliotis* in cultivated shellfish and importance of further study regarding the biodiversity and pathogenesis of *S. haliotis*.

## 1. Introduction

Genus *Shewanella* is a member of the class *Gammaproteobacteria* and comprises a group of Gram-negative, nonfermentative, and facultative anaerobic motile bacilli [1]. More than 50 species of *Shewanella* have been reported [2, 3]. They can grow anaerobically by reduction of various sulfur compounds to H_2_S, including thiosulfate and sulfite [4].

Most of the organisms detected were found in marine environments, and four of these species were commonly found in clinical specimens (*S. algae*, *S. putrefaciens*, *S. haliotis*, and *S. xiamenensis*). Among these four, *S. putrefaciens* and *S. algae* have been found increasingly in human infections [5]. *Shewanella* infection is associated with direct contact with the organism through seawater or ingestion of raw seafood [3, 6]. In addition, a subject infected by *Shewanella* spp. is usually associated with hepatobiliary disease [3, 6].

 *S*. *haliotis* was first isolated from gut microflora of abalone in 2007 [7] and was first reported to be associated with human infections in Japan [8, 9]. As a marine microorganism, it has been rarely reported in humans until recently [10, 11]. During 2012 and 2013, Liu et al. reported that *S. haliotis* is the causative organism for 5 (56%) out of 9 *Shewanella* bacteremia cases in Taiwan [12]. The increasing case reports included bacteremia, abdominal infection, and soft tissue infections [9, 13]. The risks of *Shewanella* infection caused by seafood consumption are rarely discussed and limited in the literature. In addition, the importance of ecological distribution of *S. haliotis* in the marine environment has not yet been recognized. In this study, we report the distribution of *S. haliotis* in cultivated shellfish farming in aquaculture and diverse water sources in Taiwan. The findings from this study will serve as the basis of further understanding of the relationship between different sources of *S. haliotis* for future studies.

## 2. Materials and Methods

### 2.1. Sampling Sites

According to the Fisheries Statistical Yearbook published by the Fisheries Agency of Taiwan [14], eight largest cultivated shellfish sites around Taiwan's coastal areas from twenty cultivated fishing districts were included in this study (Table 1). A total of 78 cultivated shellfish samples, including abalones (*Haliotis diversicolor*), clams (*Meretrix lusoria*), and oysters (*Crassostrea gigas*) were collected and analyzed from 2012 to 2014. The sample numbers of abalones, clams, and oysters were 12, 30, and 36, respectively. All of the abalone samples were obtained from Toucheng and Chenggong, two major townships for abalone cultivation, both located on the east coast of Taiwan. Clams and oysters farms are widely distributed on the west coast. Wuqi, Kouhu, Dongshih, Budai, Zihguan, and Fangliao were sampling sites for oysters. The clam samples were collected from Wuqi, Kouhu, Budai, Zihguan, and Fangliao.

### 2.2. Isolation of *Shewanella haliotis*

We followed the guidelines described in U.S. FDA Bacteriological Analytical Manual [15] for shellfish sampling and preparation of sample homogenate. Six samples of shellfish were selected randomly from each sampling site by hand fishing. Samples were packed into sterile plastic bags and then transported to laboratory under refrigerated condition. All the samples were stored at 0–4°C until analyzed and were examined within 6 h of collection. Shells were removed by using the sterilized appliance. One gram of sample was mixed with 100 ml of saline and then homogenized in a sterile blender. One ml of the resultant mixture was transferred and enriched in 10 ml of marine broth (BD, Sparks, USA) and incubated at 30°C for 2–5 days. After incubation, isolation was carried out by the aerobic plate count according to methods described in the Association of Official Analytical Chemists's (AOAC's) guidelines [16]. Serially, decimal dilutions of enriched bacterial culture (from 10^−1^ to 10^−6^) were prepared by transferring 1 ml of previous dilution to 9 ml of marine broth. One ml of each dilutions was spread across onto separate marine agar (BD, Sparks, USA) and then incubated at 30°C for 72 h. According to the description by Kim et al., the typical colony morphology of *S. haliotis* is circular, convex, entire margin, smooth, opaque, and pink-orange color [7]. The cells are Gram-negative bacillus (rod-shaped) and 0.5–0.7 × 2.0–4.3 *μ*m in size [7]. All eligible colonies were picked for further species identification using 16S rDNA sequence analysis. Biochemical characteristics were analyzed by API 20NE (bioMérieux, Marcy-l'Étoile, France) and API ZYM (bioMérieux, Marcy-l'Étoile, France) following the manufacturer's protocol.

### 2.3. DNA Extraction and 16S rRNA Gene Sequencing

Bacterial DNA was purified with DNeasy Blood and Tissue Kit (QIAGEN, Hilden, Germany). Two universal primers, 27F (5′-AGAGTTTGATCCTGGCTCAG-3′) and 1492R (5′-TACGGCTACCTTGTTACGACTT-3′), were performed for 16S rRNA gene amplification. Each PCR reaction panel contained 10 mM of Tris-HCl (pH 9.0), 1.5 mM of MgCl_2_, 0.2 mM of dNTP, 50 ng of chromosomal DNA template, 20 mM each of primers, and 1 U of Taq DNA polymerase in a final volume of 25 *μ*l. A full PCR cycle following a 30 s denaturation at 94°C included 25 cycles at 94°C for 1 min, 55°C for 1 min, 72°C for 2 min, and a final extension at 72°C for 5 min. All the processes mentioned above were performed on PerkinElmer GeneAmp 9600 PCR system (PerkinElmer, Norwalk, USA). Sequencing of these amplicons was completed by a sequencing company (MB MISSION BIOTECH, Taipei, Taiwan) using ABI 3730 × l DNA Analyzer (Applied Biosystems, Foster City, USA). Species identification was performed by NCBI BLAST (http://blast.ncbi.nlm.nih.gov/Blast.cgi).

### 2.4. Phylogenetic Analysis

*S. haliotis* shellfish isolates, four human clinical isolates, and one type strain, *S. haliotis* DW01 (JCM 14758), were included in the phylogenetic analysis. 16S rRNA gene sequence analysis of clinical isolates was carried out as described above. The sequence of *S. haliotis* DW01 was obtained from the NCBI database (accession number NR044134.1). The 16S rRNA gene sequences were first aligned by the Clustal W method by using the MegAlign program (DNASTAR Lasergene v. 7.1.0). The phylogenetic analysis was performed by using the neighbor-joining method [17] and constructed by MEGA 6.0 software [18] (http://www.megasoftware.net). The resultant neighbor-joining tree topology was evaluated by bootstrap analyses [19] based on 1000 bootstrap analyses. The pairwise evolutionary distances were calculated based on Kimura's two-parameter model [20].

## 3. Result

**Figure 1:**
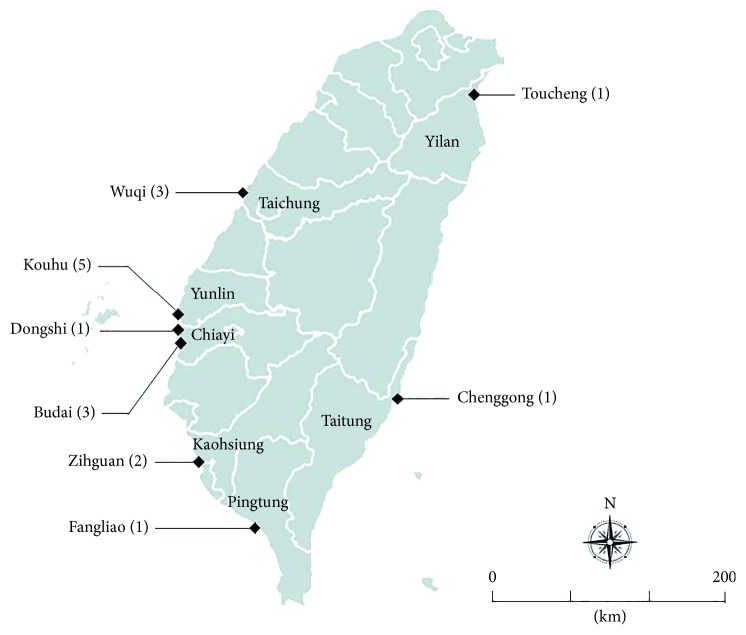
Map of sampling positions, Taiwan. Filled diamond points out the district/township name of shellfish cultivated locations. The number of obtained isolates also noted on this map, after each place name (in parenthesis).

Figure 1 shows the 8 study sampling sites, and the 17 isolates are shown in Table 1. The *S. haliotis* isolation percentages of abalones, clams, and oysters samples were 16.7% (2/12), 20% (6/30), and 25% (9/36), respectively (Table 2). All isolates grew at 4°C and 37°C and expressed typical morphological characteristics of colonies and cells. The results from API 20NE and API ZYM showed common biochemical features of clinics and shellfish isolates. Most isolates were positive for alkaline phosphatase (94%), esterase (C4) (94%), esterase lipase (C8) (94%), leucine arylamidase (94%), *α*-chymotrypsin (94%), acid phosphatase (94%), naphthol-AS-BI-phosphohydrolase (94%), and gelatinase activity (94%), and were capable of utilizing malate as a carbon source for growing (Table 3).

**Figure 2:**
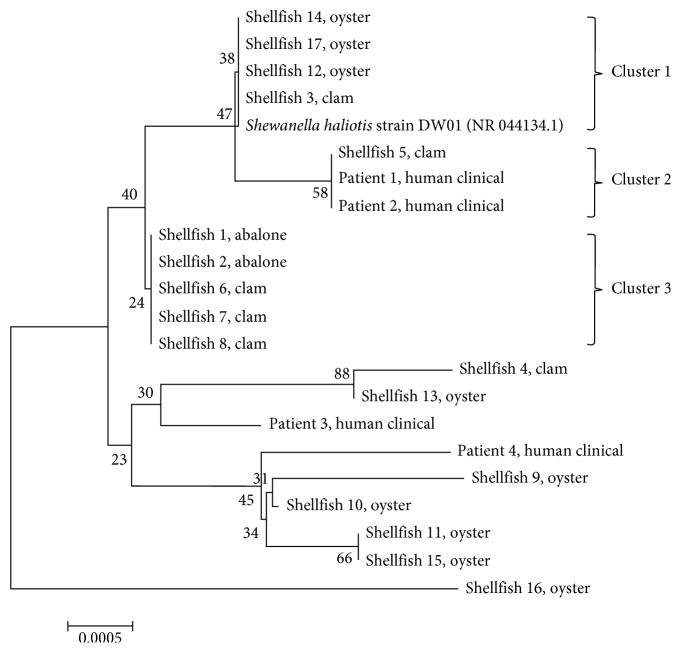
Phylogenetic tree based on 16S rDNA sequence showing the relationship between shellfish and clinical isolates of *S. haliotis*. The tree was drawn to scale with branch lengths in the same units as those of the evolutionary distances used to infer the phylogenetic tree. All positions containing gaps and missing data were eliminated. There were a total of 1320 positions in the final dataset. The scale bar indicates 0.0005 nucleotide substitutions per site.

Based on the phylogenetic tree (Figure 2), shellfish 3 of clam origin and shellfish 3, 12, 14, and 17 of oyster origin are most related with the *S. haliotis* type strain DW01 (grouped as cluster 1), followed by the isolates included in cluster 2 and cluster 3. Among all shellfish isolates, shellfish 5 (cluster 2) of clam origin possessed the closest similarity with these two clinical isolates: patient 1 and patient 2. As also shown in the phylogenetic tree, the other isolates were more diverse from these two clinical isolates. The only two abalone origin isolates, shellfish 1 and shellfish 2, were grouped together with three other clam (shellfish 6, 7, and 8) origin isolates in cluster 3. Other isolates could not represent closely related evolutionary relationships to group into any cluster. Overall, the data revealed that *S. haliotis* is a phylogenetically complex and diverse species.

## 4. Discussion

This study is the first evidence of the linkage between human and environmental isolates. The phylogenetic analysis also revealed the highly diverse nature of *S. haliotis*, which warrants further study of optimal typing scheme. The wide distribution of positive sampling sites further highlights the possibility of extensive contamination of the zoonotic pathogens.

Shellfish, such as abalones, clams and oysters, is commonly served in cuisine in Taiwan. However, the risk of *Shewanella* infection caused by seafood consumption as well as the ecological distribution of *S. haliotis* in marine environment are still unclear [21, 22]. To date, *S. haliotis* has caused several cases of soft tissue infections and bacteremia, of which most were seen in Asian countries during warm seasons [23]. Most patients lived in coastal areas and had a history of seawater contact or seafood consumption [10, 12]. The causal link between direct seawater contact and soft tissue infection has been established [3, 6]. The entry route and primary infectious loci for *S. haliotis* bacteremia, however, remains unknown. In this study, we provided results to assess the prevalence of *S. haliotis* in the distribution of aquaculture farm in Taiwan. We collected 78 samples; 21.8% (17/78) of isolates from shellfish was identified as *S. haliotis* (Tables 1 and 2). In 2007, *S. haliotis* was first isolated from the gut microflora of abalone in Korea [7] which might be misidentified as *Shewanella algae* due to biochemical test results in some *Shewanella* infection cases [10]. This implied *S. haliotis* infection might have been underestimated [10].

In our study, 17 isolates from shellfish were collected and compared with four isolates from clinics in their morphological, physiological, and biochemical characteristics. They shared a high percentage of identical biochemical characteristics in API 20NE and API ZYM tests (Table 3). In addition, 16S rDNA and sequencing analyses all revealed a rather close relationship with *S. haliotis*. As previous study pointed out that 16S sequence similarities of ≥97% should be considered as the same species [24], the 17 isolates in our study were identified as *S. haliotis* base on the 16S sequence. This close relationship was further confirmed by the phylogenetic clustering of isolates with the reference strain of *S. haliotis* DW01 (Figure.  2).

Based on the phylogenic tree, the phylogenetic relationship between different sources of *S. haliotis* is extremely multivariate. In the other words, there are many different types of *S. haliotis* present in the environment, and there might be more isolates of *S. haliotis* that can cause human diseases. The causal link between ingesting undercooked or even raw seafood, including shellfish, and the development of *Shewanella* bacteremia has been identified [25]. Byun et al. raised the concerns that this dietary habit may increase the risk to develop *Shewanella* bacteremia among people with underlying hepatobiliary diseases [10]. Of note, hepatobiliary diseases are common in Taiwan with around 8000 new hepatocellular carcinoma cases annually [26]. In addition, according to the results, we found that the profiles between clinical isolates (patient 1 and patient 2) and environmental isolates (shellfish 5) were similar, suggesting that patients may have acquired infection from cultivated shellfish. It will be helpful to study the environmental habitat and virulence factor of this group of isolates to understand why they are emerging in temperate climates, which may be an indication of the geographical distribution of *S. haliotis* in the world. Based on the analysis of 16S rRNA gene sequence for *S. haliotis* in this study, we speculate a high diversity of *S. haliotis* with various molecular types present in aquaculture environment of Taiwan.

There are some limitations of our present study, which can be readily resolved in the future. For example, the number of *S. haliotis* in shellfish could be determined by a quantitative culture or by employing quantitative real-time PCR technique. In addition, whole genomic sequencing may fill in some phylogenetic gap that could be missing based on our current strategy of 16S ribosomal RNA sequencing. Furthermore, extensive and continuous surveillance would be helpful in the future to define the evolution and spread of the disease.

## 5. Conclusion

In this study, we aimed to establish the existence of *S. haliotis* in cultivated shellfish commonly consumed, such as abalones (*Haliotis diversicolor*), clams (*Meretrix lusoria*), and oysters (*Crassostrea gigas*). Furthermore, we investigated the phylogenetic relationship between clinical and environmental isolates in Taiwan. Analysis of the data suggests the linkage between human and environmental isolates. *Shewanella* spp. have been recently associated with a number of new disease syndromes, including bacteremia and gastrointestinal infection. Patients with underlying diseases in the hepatobiliary tract seem to be susceptible to these marine pathogens. Our data support the hypothesis of *S. haliotis* as a foodborne pathogen. Further study is needed to refine our knowledge of the pathogenesis of *S. haliotis* infection.

## Figures and Tables

**Table 1:** *Shewanella haliotis* isolates, Taiwan.

Laboratory Identification	Origin of isolate	Sampling site	Identity	Accession (GenBank accession ID)
Shellfish 1	Abalone	Chenggong	99%	KF500918.1
Shellfish 2	Abalone	Toucheng	99%	KF500918.1
Shellfish 3	Clam	Kouhu	99%	JX429797.1
Shellfish 4	Clam	Budai	99%	MF928137.1
Shellfish 5	Clam	Budain	100%	MF928137.1
Shellfish 6	Clam	Wuqi	99%	JX429797.1
Shellfish 7	Clam	Wuqi	100%	KF500918.1
Shellfish 8	Clam	Wuqi	100%	KF500918.1
Shellfish 9	Oyster	Kouhu	99%	KF500918.1
Shellfish 10	Oyster	Kouhu	99%	KF500918.1
Shellfish 11	Oyster	Kouhu	99%	KF500918.1
Shellfish 12	Oyster	Kouhu	99%	JX429797.1
Shellfish 13	Oyster	Budai	99%	KF500918.1
Shellfish 14	Oyster	Zihguan	99%	JX429797.1
Shellfish 15	Oyster	Budai	99%	KF500918.1
Shellfish 16	Oyster	Fangliao	99%	KF500918.1
Shellfish 17	Oyster	Dongshih	100%	JX429797.1

**Table 2:** Statistics of *Shewanella haliotis* isolated from aquaculture shellfish in Taiwan.

Shellfish	Number of samples	Number of sites	*Shewanella haliotis*		
			Number of isolates obtained	Isolation percentage	Number of positive sampling sites
Abalone	12	2	2	16.7%	2
Clam	30	5	6	20.0%	4
Oyster	36	6^*∗*^	9	25.0%	5

^*∗*^Five out of six sites of oysters were the same as clams.

**Table 3:** Characterization of *Shewanella haliotis*.

Characteristics	% of positive reaction	
	Clinical isolates (*n*=4)	Shellfish isolates (*n*=17)
API 20 NE		
Reduction of nitrates to nitrites	75%	94%
Indole production	0%	0%
Glucose fermentation	0%	0%
Arginine dihydrolase	0%	0%
Urease	0%	0%
*β*-Glucosidase	25%	0%
Gelatinase	100%	94%
Assimilation by using		
Glucose	0%	0%
Arabinose	0%	0%
Mannose	0%	6%
Mannitol	0%	0%
N-acetyl-glucosamine	75%	82%
Maltose	0%	0%
Potassium gluconate	0%	0%
Capric acid	75%	94%
Adipic acid	0%	0%
Malate	100%	94%
Trisodium citrate	0%	0%
Phenylacetic acid	0%	0%

API ZYM		
Alkaline phosphatase	100%	94%
Esterase (C4)	100%	94%
Esterase lipase (C8)	100%	94%
Lipase (C14)	0%	0%
Leucine arylamidase	100%	94%
Valine arylamidase	0%	0%
Cystine arylamidase	0%	0%
Trypsin	0%	0%
*α*-Chymotrypsin	100%	94%
Acid phosphatase	100%	94%
Naphthol-AS-BI-phosphohydrolase	100%	94%
*α*-Galactosidase	0%	0%
*β*-Galactosidase	0%	0%
*β*-Glucuronidase	0%	0%
*α*-Glucuronidase	0%	0%
*β*-Glucosidase	0%	0%
N-Acetly-*β*-glucosaminidase	50%	53%
*α*-Mannosidase	0%	0%
*α*-Fucosidase	0%	0%

Growth at		
4°C	100%	100%
37°C	100%	100%

## Data Availability

The data used to support the findings of this study are available from the corresponding author upon request.
